# Position statement on radiopharmaceutical production for clinical trials

**DOI:** 10.1186/s41181-017-0031-y

**Published:** 2017-09-29

**Authors:** G. Bormans, A. Buck, A. Chiti, M. Cooper, J. Croasdale, M. Desruet, V. Kumar, Y. Liu, I. Penuelas, C. Rossetti, R. Schiavo, S. W. Schwarz, A. D. Windhorst

**Affiliations:** 1 Radiopharmaceutical Research KU Leuven, Leuven, Belgium; 2 University of Wurzburg, Würzburg, Germany; 3 Humanitas University and Humanitas Research Hospital, Milan, Italy; 4 Royal Liverpool University Hospital, Liverpool, UK; 5 Sandwell & West Birmingham Hospitals NHS Trust, Birmingham, UK; 6 Hospital Grenoble-Alpes, La Tronche, France; 7 Sydney Medical School, Sydney University, Camperdown, Australia; 8 European Organisation for Research and Treatment of Cancer (EORTC), Brussels, Belgium; 9 ClínicaUniversidad de Navarra, Pamplona, Spain; 10 Ospedale Niguarda, Milan, Italy; 11 Ospedale Belcolle, Viterbo, Italy; 12 Washington University School of Medicine, St. Louis, MO USA; 13 VU University Medical Center, Amsterdam, The Netherlands

**Keywords:** Radiopharmaceuticals, Regulatory, Clinical trials

## Abstract

The EU regulation 536/2014 aims to facilitate the experimental use of diagnostic radiopharmaceuticals in particular for GMP requirements and needs to be applied in EU countries. As definitely clarified by this survey, the application is still far from being completed due to national restrictions that are conflicting with the content of the above EU regulation. Although the nuclear medicine centers are obliged to be compliant with national regulatory, national authorities have to be required to work towards full application of the regulation. On the other hand, an update of 536/2014 that includes therapeutic radiopharmaceuticals would also be beneficial to a rational and safe advance of nuclear medicine.

## Background

Radiopharmaceuticals are classified as medicinal products and their production, indications and use are regulated accordingly.

In European countries, the European Commission has issued European directives setting normative standards on this subject, while the European Medicines Agency (EMA) is in charge of the review and approval of marketing authorisation applications.

In every single EU member state there is also national legislation on medicinal products that refers to the EU directives and in some instances to regulations issued by the national competent authorities. Despite their shared references to the relevant EU directives, national regulations are not exactly equivalent to each other (Decristoforo et al., 2014)

The legal conditions relating to the production of radiopharmaceuticals for clinical trials, and in particular the required compliance with the principles of good manufacturing practice (GMP), have not been clear and there has been variation between EU member states. GMP has already been implemented in some centres in Europe, including clinical centres embedded in nuclear medicine departments using cyclotrons and PET tracer production laboratories.

With the intention of overcoming the general negative effects that derived from the old Directive 2001/20 concerning clinical trials, on 27 May 2014 the European Commission issued a new regulation (No. 536/2014) (Official Journal of European Union, 2014). Since a “regulation” of the EU is to be adopted as national law without any changes, it is more stringent than a “directive”, which requires transposition into the national body of laws. Within this new clinical trial regulation, there are three relevant points regarding the preparation of radiopharmaceuticals for clinical trials:

1. Authorisation is no longer needed for the manufacture of diagnostic investigational radiopharmaceuticals for use in hospitals taking part in the same clinical trial based in the same EU member state.
2. GMP production according to EudraLex 4 is no longer required for diagnostic radiopharmaceuticals even in the case of investigational medicinal products (IMPs), but can still be imposed by local (national) regulations.
3. Simplified labelling of the primary packaging is allowed, solving the issue of the need to provide too much information on the primary packaging label.

The regulation is applicable only to diagnostic IMPs and non-IMPs; therapeutic IMPs and non-IMPs are excluded.

Regardless of the implementation of this new regulation on clinical trials, in most EU member states there is still no exemption from the general requirement for compliance with GMP even for diagnostic radiopharmaceuticals. On the basis of a recent survey conducted by the EANM in seven European countries (Spain, Italy, United Kingdom, France, Germany, the Netherlands, Belgium), it can be concluded that there are still differences between countries regarding the requirement for GMP. If radiopharmaceuticals are used in a public hospital in France, GMP is not mandatory but the hospital radiopharmacy must follow good preparation practice (which is less demanding). The same applies to Germany, where GMP rules are applied on the basis of local regional policy. In France, in both private and public hospitals, GMP is needed in relation to the starting materials for pharmaceutical use. In Italy, GMP requirements depend on the purpose of the trial, i.e. whether it is commercial or not, and this also holds true for therapeutic radiopharmaceuticals. In Belgium, the status as an IMP or a non-IMP makes a difference, as GMP is mandatory only for IMPs. In Spain, the United Kingdom and the Netherlands, GMP is always considered mandatory. Table 1 summarises the results of the survey.

**Table 1:** GMP requirements for radiopharmaceutical production for clinical trials in Europe

	IMP	Non-IMP	Diagnostic	Therapeutic	Profit	Non-profit	Public	Private
Spain	Yes	Yes	Yes	Yes	Yes	Yes	Yes	Yes
Italy	Yes	Yes	No	Yes	Yes	No	Yes	Yes
UK	Yes	Yes	Yes	Yes	Yes	Yes	Yes	Yes
France	Yes	Yes	Yes	Yes	Yes	Yes	No	Yes
Germany	Yes	Yes	Yes	Yes	Yes	Yes	No	Yes
The Netherlands	Yes	Yes	Yes	Yes	Yes	Yes	Yes	Yes
Belgium	Yes	No	*YES*	*YES*	*YES*	*YES*	*YES*	*YES*

Preparation of radiopharmaceuticals for clinical trials is a crucial activity for Nuclear Medicine community. In fact, nuclear medicine departments are involved in clinical trials on both diagnostic and therapeutic radiopharmaceuticals, and there is an emerging and growing demand for new radiopharmaceuticals following the experimental phase for registration. This is particularly true for radiopharmaceuticals with short half-lives that are suitable for use with positron emission tomography scanners. However, the fact that not all clinical centres in Europe are qualified for GMP preparation seriously limits the use of such radiopharmaceuticals for clinical trials. Correct implementation of Regulation 536/2014 could partially overcome this hurdle, at least for diagnostic radiopharmaceuticals to be employed in clinical trials.

Alternatively, one might advocate the implementation of GMP requirements only in a few selected centres. While such a centralized radiopharmacy approach could work for radiopharmaceuticals with long half-lives (e.g. > 1 day), it could not be easily applied for radiopharmaceuticals with short half-lives (e.g. ≥ 2 h) and would never be feasible for those with very short half-lives (e.g.a few minutes).

## Conclusions

In conclusion, the EU regulation 536/2014 aims to facilitate the experimental use of diagnostic radiopharmaceuticals in particular regarding GMP requirements and needs to be applied in EU countries. As definitely clarified by this survey, the application is still far from being completed due to national restrictions that are conflicting with the content of the above EU regulation. Although the nuclear medicine centres are obliged to be compliant with national regulatory, national authorities have to be required to work towards full application of the new regulation, in particular concerning GMP production for diagnostic radiopharmaceuticals in clinical trials. On the other hand, an update of 536/2014 that includes therapeutic radiopharmaceuticals could be matter of fruitful open discussion involving EMA and European Commission for a rational and safe advance of nuclear medicine.
